# Supplementary material for: Complete mitochondrial genome sequences of the northern spotted owl (Strix occidentalis caurina) and the barred owl (Strix varia; Aves: Strigiformes: Strigidae) confirm the presence of a duplicated control region
Source: PeerJ. 2017 Oct 10;5:e3901. doi: 10.7717/peerj.3901 (PMC5639871; doi:10.7717/peerj.3901)
Supplement: Supplemental Information 1 — Supplementary details of methodology and Figures S1–S3. [file peerj-05-3901-s001.pdf]

Supplementary Materials for:

Complete mitochondrial genome sequences of the northern spotted owl (*Strix occidentalis caurina*) and the barred owl (*Strix varia*; Aves: Strigiformes: Strigidae) confirm the presence of a duplicated control region

Zachary R. Hanna,[1,2,3,4,7] James B. Henderson,[3,4] Anna B. Sellas,[4,5] Jérôme Fuchs,[3,6] Rauri C. K. Bowie,[1,2] John P. Dumbacher[3,4]

[1] Museum of Vertebrate Zoology, University of California, Berkeley, Berkeley, California, United States of America

[2] Department of Integrative Biology, University of California, Berkeley, Berkeley, California, United States of America

[3] Department of Ornithology & Mammalogy, California Academy of Sciences, San Francisco, California, United States of America

[4] Center for Comparative Genomics, California Academy of Sciences, San Francisco, California, United States of America

[5] Chan Zuckerberg Biohub, San Francisco, California, United States of America

[6] UMR 7205 Institut de Systématique, Evolution, Biodiversité, CNRS, MNHN, UPMC, EPHE, Sorbonne Universités, Muséum National d'Histoire Naturelle, Paris, France

[7] Corresponding Author:

Zachary Hanna

Email address: zachanna@berkeley.edu

1 Supplementary Methods

1.1 Initial S. o. caurina assembly

1.1.1 BLATq version 1.0.2 (Henderson & Hanna, 2016a) used to align our 150 bp read 1 sequences from the Nextera700bp library (Hanna et al., 2017) to the *Ninox novaeseelandiae* mitochondrial genome (GenBank accession AY309457) using default BLAT parameters other than "-stepSize=5 -repMatch=100000 -out=blast8".

1.1.2 excerptByIds version 1.0.2 (Henderson & Hanna, 2016b) used, to extract the pairs of reads that had BLATq hits to the *Ninox novaeseelandiae* mitochondrial genome.

1.1.3 SOAPdenovo2 version 2.04 (Luo et al., 2012) used with default settings except "SOAPdenovo-127mer all -m 127 -R". In our configuration file for this assembly we used the default minimum alignment length between a read and contig (32 for paired-end reads) and the default minimum pair number cutoff (3 for paired-end reads) and set the reads to be used for the assembly of both contigs and scaffolds.

1.1.4 Web version of the NCBI BLAST+ version 2.2.29 tool BLASTn (Altschul et al., 1990; Zhang et al., 2000; Morgulis et al., 2008; Camacho et al., 2009) with default parameters to search the NCBI nucleotide collection (Johnson et al., 2008; Boratyn et al., 2013; NCBI Resource Coordinators, 2015; Benson et al., 2015).

1.1.5 GNU Grep version 2.16 (Free Software Foundation, 2014) used to search the trimmed and merged reads from lane 1 and 2 of the Nextera550bp library for reads that matched the assembled sequence of $tRNA^{Phe}$ or $tRNA^{Thr}$.

1.1.6 We found 3 reads that spanned $tRNA^{Phe}$ (1 of which was a merged read pair (Hanna et al., 2017)) and combined them using the Geneious version 9.1.4 (Kearse et al., 2012;

Biomatters, 2016) *de novo* assembler. The resulting contig contained spanned from the control region through $tRNA^{Phe}$ and into *12S*.

1.1.7 PRICE version 1.2 (Ruby, Bellare & DeRisi, 2013; Ruby, 2014) used with the assembled contig spanning $tRNA^{Phe}$ as the initial contig and the parameters "-spf <merged_reads> 300 600 -icf <initial_contig.fasta> 1 1 5 -mol 25 -mpi 85 -MPI 80 -nc 60 -lenf 40 5 -lenf 90 10 -a 10 -target 85 1 1 1 -maxHp 25 -o <output.fa> -o <output.priceq>". The "merged_reads" parameter referred to the merged overlapping sequences from lane 1 of the Nextera550bp library.

1.1.8 BLATq version 1.0.2 (Henderson & Hanna, 2016a) to align our 150 bp read 1 sequences from the Nextera700bp library to the assembly output by PRICE using default settings other than "-stepSize=5 -repMatch=100000 -out=blast8".

1.1.9 excerptByIds version 1.0.2 (Henderson & Hanna, 2016b) to extract the read 2 of the paired-end sequences corresponding to the aligned read 1 sequences.

1.1.10 PRICE assembly with the same initial contig as before, but with more sequence data, including the merged overlapping sequences from both lane 1 and 2 of the Nextera550bp library and the matching paired-end sequences from the Nextera700bp library. We used the default PRICE assembly settings with the following exceptions: "-fp <Nextera700bp read 1 matches> <Nextera700bp read 2 matches> 809 -spf <Nextera550bp lane 1 merged reads> 300 600 -spf <Nextera550bp lane 2 merged reads> 300 600 -icf <initial_contig.fasta> 1 1 5 -mol 25 -mpi 85 -MPI 80 -nc 60 -lenf 40 5 -lenf 90 10 -a 10 -target 85 1 1 1 -maxHp 25 -o <output.fa> -o <output.priceq>".

1.1.11 MITOS WebServer version 605 (Bernt et al., 2013) specifying "genetic code = 02 - Vertebrate".

1.1.12 Tandem Repeats Finder version 4.07b (Benson, 1999, 2012) used with default options.

1.2 S. o. caurina Sanger sequencing assembly confirmation

1.2.1 We isolated genomic DNA using a DNeasy Blood & Tissue Kit (Qiagen, U.S.A.). Polymerase chain reaction (PCR) conditions for primers 17589F and 41R included an initial denaturation at 94°C for 1 min; then 30 cycles at 94°C for 30 s, 60°C for 30 s, and 72°C for 2 min; and a final extension at 72°C for 7 min. We then sequenced both ends of the PCR-amplified fragment using BigDye terminator chemistry (Applied Biosystems, U.S.A.) on an ABI 3130xl automated sequencer (Applied Biosystems, U.S.A.).

1.2.2 Polymerase chain reaction (PCR) conditions for primers 17572F and 41R were the same as for primers 17589F and 41R (initial denaturation at 94°C for 1 min; then 30 cycles at 94°C for 30 s, 60°C for 30 s, and 72°C for 2 min; and a final extension at 72°C for 7 min). We sequenced the PCR products using BigDye terminator chemistry (Applied Biosystems, U.S.A.) on an ABI 3130xl automated sequencer (Applied Biosystems, U.S.A.).

1.2.3 We used the Geneious mapper through the "map to reference" function with default options other than sensitivity set to "highest sensitivity / slow" to align the edited Sanger-derived sequences to the 19,946 nt preliminary mitochondrial genome assembly.

1.2.4 Polymerase chain reaction (PCR) conditions for primers cytb-F1 and 17122R included an initial denaturation at 94°C for 3 min; then 35 cycles at 94°C for 30 s, 50°C for 30 s, and 72°C for 2 min; and a final extension at 72°C for 10 min.

1.3 S. o. caurina final assembly

1.3.1 We removed scaffold-3674 from the draft whole nuclear genome assembly (Hanna et al., 2017) using the "filterbyname.sh" tool in the BBMap tool suite version 36.02 (Bushnell,

2016) and replaced it with the 19,948 nt mitochondrial genome assembly from our targeted assembly methodology. When referring to specific scaffolds here and in the manuscript, we have inserted a dash ("-") between the word "scaffold" and the scaffold number for legibility. These dashes are not present in any of the assembly data files. Thus, "scaffold-3674" referenced in the manuscript will appear as "scaffold3674" in the assembly and other associated files.

1.3.2 We aligned all filtered Illumina sequences to this new draft reference genome using bwa version 0.7.13-r1126 (Li, 2013a) using default options except parameters "bwa mem -M". We separately aligned paired-end and unpaired reads. We then merged the paired-end and unpaired read alignments using the Picard version 2.2.4 (http://broadinstitute.github.io/picard) function MergeSamFiles and sorted them using the Picard function SortSam, employing default settings for both tools. We next marked duplicate reads (both PCR and optical) using the Picard function MarkDuplicates, employing default settings.

1.3.3 We then filtered out duplicate reads, low quality alignments, secondary alignments, and alignments where both reads of a pair did not align to the mitochondrial assembly. We used Samtools to filter out duplicate reads marked by Picard using the Samtools parameters "-F 0x400". We next used Samtools to filter out alignments of quality less than 10 with the parameters "-q 10". We then filtered out secondary alignments with the Samtools parameters "-F 0x100". We then used Samtools with GNU Awk (GAWK) version 4.0.1 (Free Software Foundation, 2012) to filter out paired reads where one of the reads mapped to a different contig/scaffold than the mitochondrial genome using

parameters “samtools view -h <input.bam> | awk '$7 == "=" || $7 == "*" || $1 ~ "^@"' | samtools view -Sb - > <output.bam>”.

1.3.4 We used Samtools version 1.3 with HTSlib 1.3.1 (Li et al., 2009, 2015) with GAWK version 4.0.1 (Free Software Foundation, 2012) to filter out all aligned sequences less than 300 nt using parameters “samtools view -h <input.bam> | awk '$1 ~ "^@"' || length($10) >= 300’ | samtools view -Sb - > <output.bam>”.

1.3.5 We visually inspected all sites where there was lower coverage and any hint of disagreement between reads and, except in the case of CR1 and CR2 where we relied on the Sanger-derived sequence data, decided in favor of majority evidence, which matched our preliminary assembly at all sites, providing confirmation of our assembly methodology.

1.4 S. o. caurina final annotation

1.4.1 MITOS WebServer version 806 (Bernt et al., 2013) specifying “genetic code = 02 - Vertebrate”.

1.4.2 Web version of Tandem Repeats Finder version 4.09 (Benson, 1999, 2016) employing default options.

1.4.3 We used bioawk version 1.0 (Li, 2013b) and GAWK version 4.0.1 (Free Software Foundation, 2012) to find goose hairpin sequences by searching for 7 C’s followed by 1 to 3 D nucleotides (A, G, or T) followed by 7 C’s in a FASTA format file (Pearson & Lipman, 1988) with each control region input as a separate entry with the command “bioawk -c fastx '{print $seq}' $1 | awk '{pos=match($0, /CCCCCCC[AGT]{1,3}CCCCCCC/);if(pos){print pos}}'”.

1.4.4 We used the NCBI BLAST+ version 2.4.0 (Altschul et al., 1990; Zhang et al., 2000; Morgulis et al., 2008; Camacho et al., 2009) tool “makeblastdb” with options “-parse_seqids -dbtype nucl” to create a database of the scaffold-3674 gene sequences and then the tool “blastn” with default options except “-outfmt 6” to align the targeted assembly gene sequences against this database.

1.4.5 We aligned the primers we developed to amplify control region 2 and the N1 primer used by Barrowclough, Gutierrez & Groth (1999) to amplify a portion of control region 1 to the final assembly using Geneious version 9.1.4 mapper through the “map to reference” function (Kearse et al., 2012; Biomatters, 2016) with default options other than using the sensitivity set at “highest sensitivity / slow”. We determined the position in the final assembly of the D16 primer used by Barrowclough, Gutierrez & Groth (1999) to amplify a portion of control region 1 by using the Geneious version 9.1.4 *de novo* assembler (Kearse et al., 2012; Biomatters, 2016) with default parameters other than setting sensitivity at “highest sensitivity / slow” to assemble control region 1 with the D16 primer.

1.4.6 We performed a multiple alignment of control regions 1 and 2 using the Geneious version 9.1.4 (Kearse et al., 2012; Biomatters, 2016) implementation of the MUSCLE version 3.8.425 (Edgar, 2004) aligner with default options.

1.4.7 We used the web version of NCBI’s BLAST+ Version 2.5.0 tool BLASTN (Altschul et al., 1990; Zhang et al., 2000; Morgulis et al., 2008; Camacho et al., 2009) with default parameters to search the NCBI nucleotide collection (Johnson et al., 2008; Boratyn et al., 2013; NCBI Resource Coordinators, 2015; Benson et al., 2015) for sequences similar to

control regions 1 and 2 in order to assess whether published control region sequences of related species are more similar to control region 1 or 2.

1.4.8 As a result of these searches, we aligned the primers used by Omote et al. (2013) to amplify the control region in *Strix uralensis* to the final assembly using Geneious version 9.1.4 mapper with the "map to reference" function (Kearse et al., 2012; Biomatters, 2016) with default options other than using the Geneious mapper with sensitivity set at "highest sensitivity / slow".

1.5 Nuclear pseudogenation of S. o. caurina mitochondrial genes

1.5.1 We first used the NCBI BLAST+ version 2.4.0 tool BLASTN (Altschul et al., 1990; Zhang et al., 2000; Morgulis et al., 2008; Camacho et al., 2009) to align the final *S. o. caurina* mitochondiral genome assembly to the draft nuclear genome assembly (Hanna et al., 2017) using default parameters other than "-outfmt 6".

1.5.2 We used GAWK version 4.0.1 (Free Software Foundation, 2012) to remove all alignments to scaffold3674, which was the assembly of the mitochondrial genome in the draft nuclear genome assembly.

1.5.3 We next used GAWK version 4.0.1 (Free Software Foundation, 2012) to reformat the BLAST output into a BED (Browser Extensible Data) formatted file (http://genome.ucsc.edu/FAQ/FAQformat#format1) with the parameters "cat <filtered_BLAST_file> | awk 'BEGIN {OFS = "\t"} {print "Strix_Occidentalis",$7,$8,$2,$9,$10,$12}' | awk 'BEGIN {OFS = $"\t"} {if ($3<$2) print $1,$3,$2,$4,$5,$6,7; else print $0}'".

1.5.4 We used BEDTools version 2.26.0 tool "intersect" (Quinlan & Hall, 2010) to produce a BED file of the intersection of the BED-formatted BLAST output with the BED file

output from the MITOS annotation of the final mitochondrial genome assembly using the parameters "-a <BED-formatted BLAST output file> -b <MITOS annotation BED file> -wo". We then used the output of the intersection to determine the mitochondrial genes spanned by each *Numt*.

1.6 Strix varia sample CAS95964

1.6.1 We extracted genomic DNA using a DNeasy Blood & Tissue Kit (Qiagen, U.S.A.). We used 50 ng genomic DNA to prepare a whole-genome library using a Nextera DNA Sample Preparation Kit (Illumina, U.S.A.). After tagmentation, we cleaned the reaction with a DNA Clean & Concentrator -5 kit (Zymo Research, U.S.A.). We amplified the reaction with 5 cycles of PCR using a KAPA Library Amplification kit (KAPA Biosystems, U.S.A.) and then cleaned the reaction with a DNA Clean & Concentrator -5 kit (Zymo Research, U.S.A.). We used Dye-Free, 1.5% agarose, 250-1,500 base pair (bp) cassette on a BluePippin (Sage Science, U.S.A.) to select library fragments in the size range of 534-634 bp, which, after subtracting the 134 bp of adapters, corresponded to selecting an average insert size of 450 bp. We next performed a real-time PCR (rtPCR) using a KAPA Real-Time Library Amplification Kit (KAPA Biosystems, U.S.A.) on a CFX96 Touch Real-Time PCR Detection System (Bio-Rad, U.S.A.) to further amplify the library with 9 cycles PCR. We then cleaned the PCR products with a DNA Clean & Concentrator -5 kit (Zymo Research, U.S.A.). We lastly assessed the library fragment size distribution with a 2100 BioAnalyzer (Agilent Technologies, U.S.A.) and the concentration of double-stranded DNA material with a Qubit 2.0 Flurometer (Invitrogen, U.S.A.). Due to the presence of small peaks in the BioAnalyzer trace, we further cleaned the library using 0.6X Agencourt AMPure XP (Beckman Coulter, U.S.A.) magnetic

beads. We obtained approximately one lane of 100 bp paired-end data sequenced in an indexed pool using a 2-lane flow cell with a HiSeq PE Rapid Cluster Kit and a 200 cycle HiSeq Rapid SBS Kit v1 on a HiSeq 2500 (Illumina, U.S.A.). The raw sequences are available upon request.

1.6.2 We performed adapter and quality trimming of the sequence data using Trimmomatic version 0.30 (Bolger, Lohse & Usadel, 2014) with the following options: "ILLUMINACLIP:<FASTA format file of Illumina adapter sequences>:2:30:7 LEADING:3 TRAILING:3 SLIDINGWINDOW:4:15 MINLEN:36".

1.6.3 For use in only the SOAPdenovo2 assembly, we trimmed the sequences using a different set of parameters and performed error-correction of the sequences. We performed adapter and quality trimming using Trimmomatic version 0.30 (Bolger, Lohse & Usadel, 2014) with the following options: "ILLUMINACLIP:<FASTA format file of Illumina adapter sequences>:2:30:7 LEADING:3 TRAILING:3 SLIDINGWINDOW:4:15 MINLEN:36 HEADCROP:12". We merged into a single file all of the single reads that resulted after their pair was dropped in the trimming process. Then we used the k-mer-based error corrector in the SOAPdenovo2 toolkit, SOAPec version 2.01 (Luo et al., 2012), to correct sequence errors. We first used the KmerFreq_HA tool to create a kmer frequency spectrum with default options except "-k 27", which indicate that we used a kmer size of 27 for creating the spectrum. We then used the Corrector_HA tool along with the kmer frequency spectrum that we created to correct all of our trimmed reads using default options except "-k 27 -r 36", which indicate that we used a kmer size of 27 for the error correction and kept trimmed reads as short as 36 bp.

1.7 Strix varia sample CMCB41533

1.7.1 We obtained tissue from a *S. varia* collected in Hamilton County, Ohio, United States of America (CNHM<USA-OH>:ORNITH:B41533; Table 1), hereafter "CMCB41533", which is well outside of the zone of contact of *S. varia* and *S. occidentalis caurina* (Haig et al., 2004). We obtained paired-end Illumina sequence data from a genomic library constructed, sequenced, and the data processed as described in (Hanna et al., 2017). The raw sequences are available from NCBI (SRA run accessions SRR5428115, SRR5428116, and SRR5428117).

1.8 Strix varia mitochondrial genome assembly

1.8.1 We generated the mitochondrial genome assembly of the *S. varia* sample CMCB41533 by building a succession of assemblies that contributed information to the final assembly from which we extracted the gene sequences. We used assemblies of sample CAS95964 to inform the process, but, as we had more sequence data for sample CMCB41533, we chose to only produce a final assembly for this sample.

1.9 Assembly of Strix varia ContigInput1

1.9.1 We used bwa version 0.7.13-r1126 (Li, 2013a) with default options other than parameters "bwa mem -M". We separately aligned paired-end and unpaired reads.

1.9.2 We merged the paired-end and unpaired read alignments using the Picard version 2.2.4 (http://broadinstitute.github.io/picard) function MergeSamFiles and sorted them using the Picard function SortSam, employing default settings for both tools. We next marked duplicate reads (both PCR and optical) using the Picard function MarkDuplicates, employing default settings.

1.9.3 We filtered the alignment file for only alignments to the final mitochondrial genome assembly using Samtools version 1.3 with HTSlib 1.3.1 (Li et al., 2009, 2015). We then

used Samtools to filter out duplicate reads marked by Picard using the Samtools parameters “-F 0x400”. We next used Samtools to filter out alignments of quality less than 10 with the parameters “-q 10”. We then filtered out secondary alignments with the Samtools parameters “-F 0x100”. We then used Samtools with GNU Awk (GAWK) version 4.0.1 (Free Software Foundation, 2012) to filter out paired reads where one of the reads mapped to a different contig/scaffold than the mitochondrial genome using parameters “samtools view -h <input.bam> | awk '$7 == "=" || $7 == "*" || $1 ~ "^@"' | samtools view -Sb - > <output.bam>”.

1.9.4 We visualized the alignment across the reference sequence in Geneious version 9.1.4 (Kearse et al., 2012; Biomatters, 2016). We used Geneious to generate a consensus sequence with default parameters for the alignment to the mitochondrial genome.

1.9.5 We extracted 3 sequences from this consensus sequence based on the *S. o. caurina* annotations to give a 142 nt fragment spanning from nucleotide 5 of $tRNA^{Phe}$ part way into *12S*; a longer, 844 nt fragment spanning from nucleotide 4 of $tRNA^{Phe}$ part way into *12S*; and a 1,042 nt fragment spanning from nucleotide 142 of *cyt b* part way into $tRNA^{Thr}$. We then used these extracted sequences as three separate seed contigs in an assembly using PRICE version 1.2 (Ruby, Bellare & DeRisi, 2013; Ruby, 2014). We used the trimmed CMCB41533 paired read 1 and 2 sequences as the sequence data for this run. Our PRICE assembly parameters were the defaults other than the following “-fp <read 1 paired sequences> < read 2 paired sequences> 466 -icf <initial_contig.fasta> 1 1 5 -mol 30 -mpi 90 -MPI 85 -nc 60 -a 8 -target 85 1 1 1 -maxHp 25 -o <output.fa> -o <output.priceq>”.

1.10 Assembly of ContigInput2 - CAS95964

1.10.1 We first used SOAPdenovo2 version 2.04 (Luo et al., 2012) to assemble all of the trimmed, error-corrected CAS95964 sequences, employing default parameters other than the options "SOAPdenovo all -m 63 -R". In our configuration file for this assembly we used the default minimum alignment length between a read and contig (32) and the default minimum pair number cutoff (3) for both the paired-end and single-end reads. We set the paired-end reads to be used for the assembly of both contigs and scaffolds and the single-end reads for use only in contig assembly. We input both the paired-end and the single-end reads with a rank of 1 and set the average insert size as 446 bp. This produced a contig of length 15,019 nt.

1.10.2 We extended the contig using PRICE version 1.2 (Ruby, Bellare & DeRisi, 2013; Ruby, 2014). We employed the trimmed (and not error-corrected) CAS95964 paired read 1 and 2 sequences as the sequence data for this run. We used the 15,019 nt contig output from the SOAPdenovo2 run above as the initial contig. Our PRICE assembly parameters were the defaults other than the following "-fp <read 1 paired sequences> <read 2 paired sequences> 446 -icf <initial_contig.fasta> 1 1 5 -mol 25 -mpi 85 -MPI 80 -nc 60 -lenf 40 5 -lenf 90 10 -a 10 -target 85 1 1 1 -maxHp 25 -o <output.fa> -o <output.priceq>".

1.11 Assembly of ContigInput2 - CMCB41533 PRICE

1.11.1 We employed the trimmed CMCB41533 paired read 1 and 2 sequences as the sequence data for this run. We used the 16,652 nt contig output from the CAS95964 run above as the initial contig. Our PRICE version 1.2 (Ruby, Bellare & DeRisi, 2013; Ruby, 2014) assembly parameters were the defaults other than the following "-fp <read 1 paired sequences> < read 2 paired sequences> 350 -icf <initial_contig.fasta> 1 1 5 -mol 25 -mpi

85 -MPI 80 -nc 60 -lenf 40 5 -lenf 90 10 -a 10 -target 85 1 1 1 -maxHp 25 -o <output.fa> -o <output.priceq>".

1.12 Final Strix varia assembly

1.12.1 We used PRICE version 1.2 (Ruby, Bellare & DeRisi, 2013; Ruby, 2014) the 9,690 nt ContigInput1 contig output from cycle 16 of the initial CMCB41533 PRICE run and the 17,073 nt ContigInput2 as the initial contigs. We used the CMCB41533 paired read 1 and 2 sequences as well as the unpaired read 1 sequences that lost their mate as a result of quality trimming for the sequence data input for this assembly. Our PRICE assembly parameters were the defaults other than the following "-fp <read 1 paired sequences> < read 2 paired sequences> 400 -spf <read 1 unpaired> 110 200 -icf <initial_contig.fasta> 1 1 5 -mol 25 -mpi 85 -MPI 80 -nc 60 -lenf 40 5 -lenf 90 10 -a 24 -target 85 1 1 1 -maxHp 25 -o <output.fa> -o <output.priceq>".

1.12.2 PCR conditions for primers cytb-F1 and 17122R included an initial denaturation at 94°C for 3 min; then 35 cycles at 94°C for 30 s, 53°C for 30 s, and 72°C for 2 min; and a final extension at 72°C for 10 min.

1.12.3 PCR conditions for primers ND6-ext1F and 12S-ext1R included an initial denaturation at 94°C for 3 min; then 35 cycles at 94°C for 30 s, 54°C for 30 s, and 72°C for 2 min; and a final extension at 72°C for 10 min.

1.12.4 We annotated the PRICE assembly using the MITOS WebServer version 605 (Bernt et al., 2013) specifying "genetic code = 02 - Vertebrate".

1.13 Comparison of Strix occidentalis and Strix varia mitochondrial genes

1.13.1 MAFFT version 7.305b (Katoh et al., 2002; Katoh & Standley, 2013; Katoh, 2016) used with the default options other than parameters "--auto --clustalout".

1.13.2 trimAl version 1.4.rev15 (Capella-Gutiérrez, Silla-Martínez & Gabaldón, 2009; Capella-Gutiérrez & Gabaldón, 2013) with default options other than "-mega" used to convert the alignments to MEGA format (Kumar, Tamura & Nei, 1994; Kumar, Stecher & Tamura, 2016).

1.13.3 MEGA version 7.0.18 (Kumar, Stecher & Tamura, 2016) used to calculate the p-distance (an uncorrected pairwise distance that is the proportion of nucleotide sites at which two sequences are different obtained by dividing the number of differences by the total number of nucleotide sites) between *S. occidentalis caurina* and *S. varia* for each gene with all alignment positions with gaps or missing data removed from the analysis.

1.13.4 MEGA version 7.0.18 (Kumar, Stecher & Tamura, 2016) used to calculate for each gene the pairwise distance corrected by the Tamura-Nei model (TN93) of DNA sequence evolution (Tamura & Nei, 1993) with rate variation among sites modeled using a gamma distribution with shape parameter = 1, differences in the composition bias of sequences considered in the comparisons (Tamura & Kumar, 2002), and with all alignment positions with gaps or missing data removed from the analysis.

1.13.5 We weighted each distance by the length of the gene alignment from which it was derived as a proportion of the total alignment length across all gene alignments and calculated a weighted average pairwise distance across all of the genes.

2 Supplementary Figures

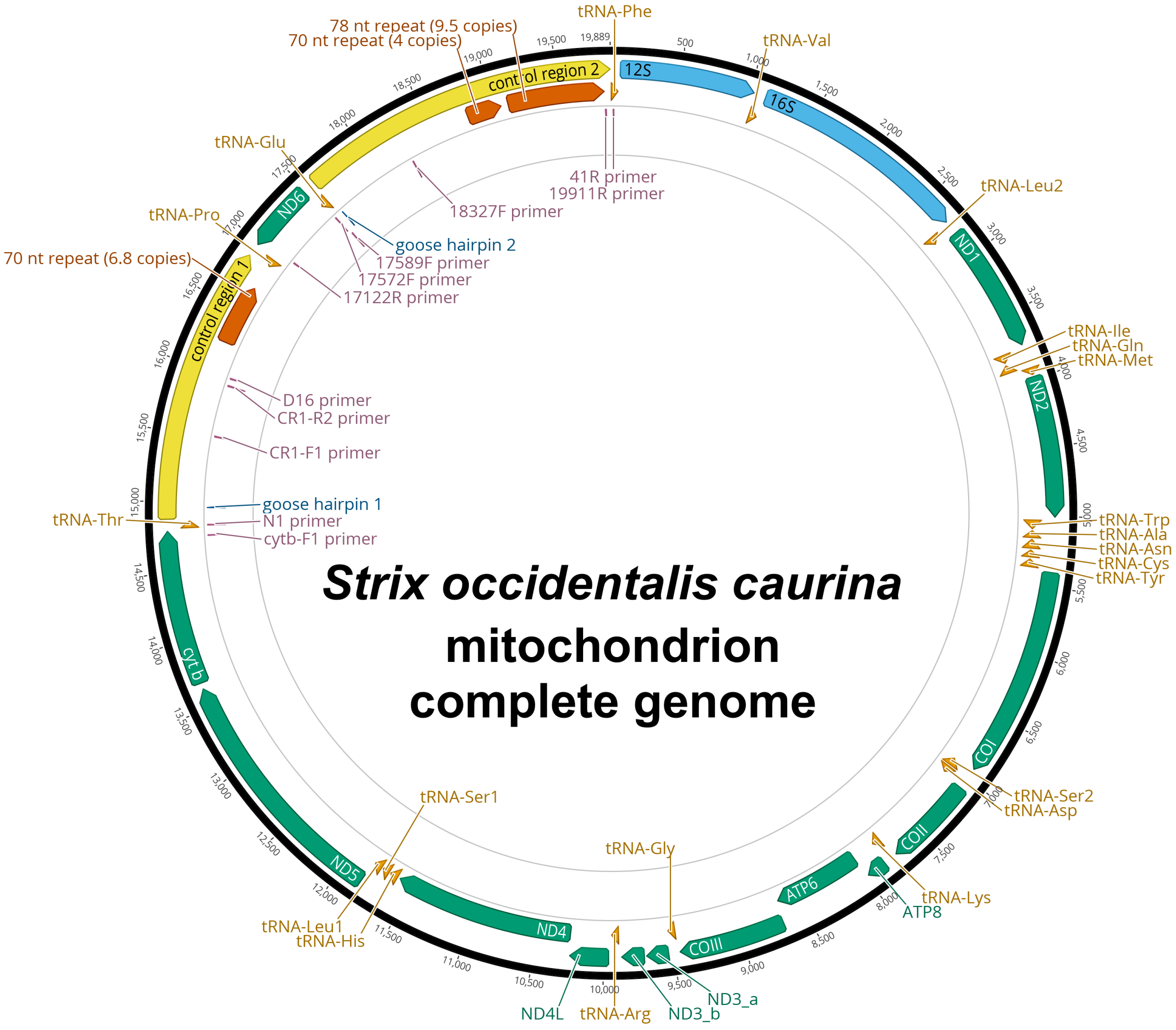

Figure S1. Complete genome of the *Strix occidentalis caurina* mitochondrion.

This is a graphical representation of the annotated complete genome of the northern spotted owl (*Strix occidentalis caurina*) mitochondrion. We have color-coded the various annotations, including genes for rRNA in sky blue, tRNA genes in orange, and all other genes in bluish green. The control regions are in yellow and the goose hairpin for each control region is depicted in blue. The locations of the primers we developed to amplify control regions 1 and 2 as well as the N1 and D16 primers used by Barrowclough et al. (1999) to amplify a portion of control region 1 are in reddish purple. The reverse complement versions of primers used ("-RC"

versions) are not shown. Regions with repetitive motifs are in vermillion. The base numbers around the perimeter of the figure are in nucleotides. We used Geneious version 9.1.4 (Kearse et al., 2012; Biomatters, 2016) to construct this figure.

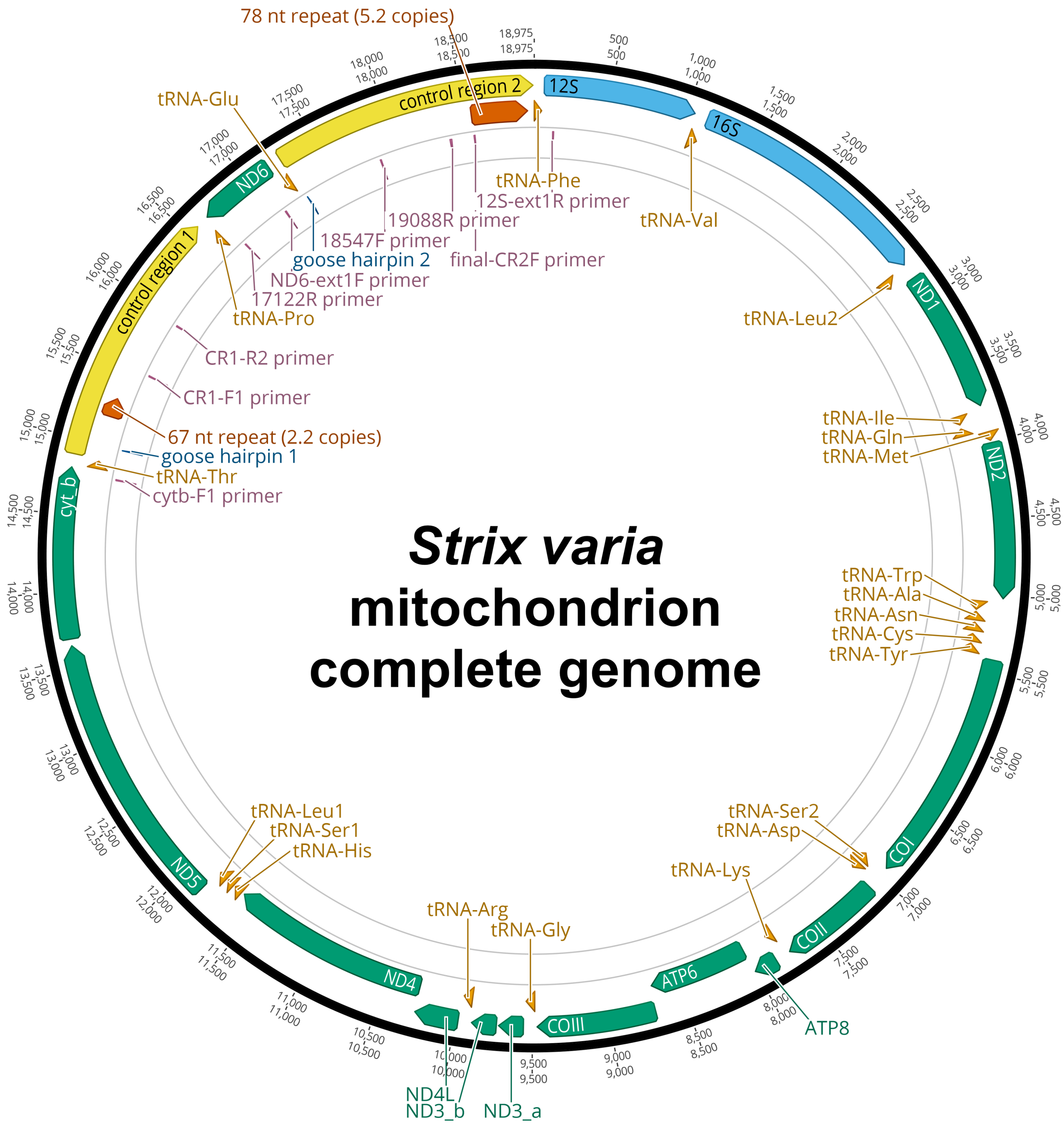

Figure S2. Complete genome of the *Strix varia* mitochondrion.

This is a graphical representation of the annotated complete genome of the barred owl (*Strix varia*) mitochondrion. We have color-coded the various annotations, including genes for rRNA in sky blue, tRNA genes in orange, and all other genes in bluish green. The control regions are in yellow and the goose hairpin for each control region is depicted in blue. The

locations of the primers we developed to amplify control regions 1 and 2 are in reddish purple. The reverse complement versions of primers used (“-RC” versions) are not shown. Regions with repetitive motifs are in vermillion. The base numbers around the perimeter of the figure are in nucleotides. We used Geneious version 9.1.4 (Kearse et al., 2012; Biomatters, 2016) to construct this figure.

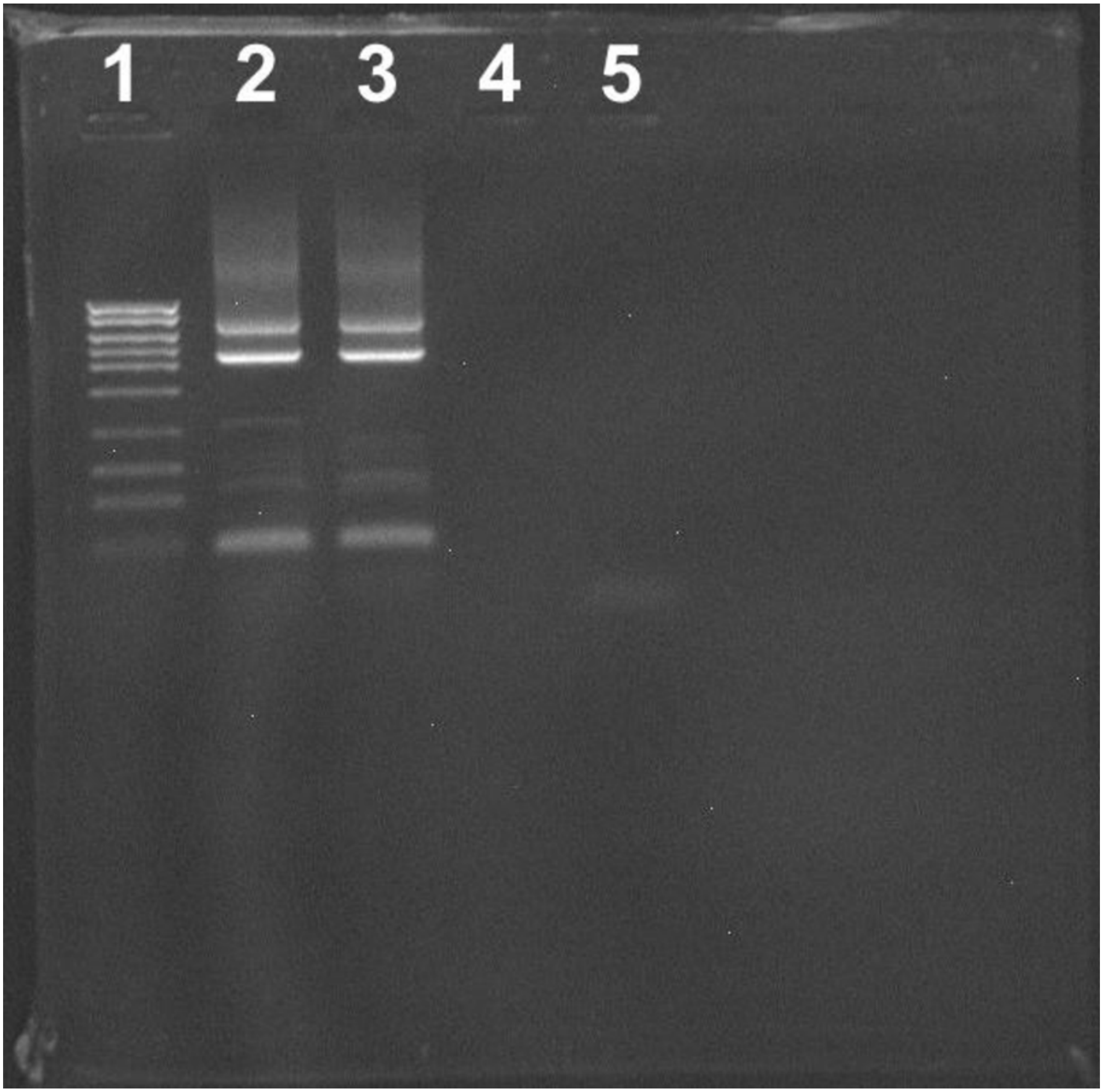

Figure S3. *Strix occidentalis caurina* CR1 PCR-amplification products.

This photograph of an agarose gel displays the lengths of the two products of PCR-amplification of the *S. o. caurina* CR1 using primers cyb-F1 and 17122R. In lane 1 we loaded Fisher BioReagents exACTGene DNA Ladder (Cat. No. BP2576100; Fisher Scientific) the ten bands of which were of lengths 5,000; 4,000; 3,000; 2,500; 2,000; 1,500; 1,000; 700; 500; and

300 nt. Lanes 2 and 3 contained independent PCR replicates of CR1 amplification products. Lane 4 was blank. In lane 5 we loaded the negative control for the PCR.

3 References

Altschul SF., Gish W., Miller W., Myers EW., Lipman DJ. 1990. Basic local alignment search tool. *Journal of Molecular Biology* 215:403–410. DOI: 10.1016/S0022-2836(05)80360-2.

Barrowclough GF., Gutierrez RJ., Groth JG. 1999. Phylogeography of Spotted Owl (*Strix occidentalis*) Populations Based on Mitochondrial DNA Sequences: Gene Flow, Genetic Structure, and a Novel Biogeographic Pattern. *Evolution* 53:919–931. DOI: 10.2307/2640731.

Benson G. 1999. Tandem repeats finder: a program to analyze DNA sequences. *Nucleic Acids Research* 27:573–580. DOI: 10.1093/nar/27.2.573.

Benson G. 2012. Tandem Repeats Finder. Version 4.07b. [Accessed 2016 Oct 1]. Available from: https://tandem.bu.edu/trf/trf.html.

Benson G. 2016. Tandem Repeats Finder. Version 4.09. [Accessed 2016 Oct 1]. Available from: https://tandem.bu.edu/trf/trf.html.

Benson DA., Clark K., Karsch-Mizrachi I., Lipman DJ., Ostell J., Sayers EW. 2015. GenBank. *Nucleic Acids Research* 43:D30–D35. DOI: 10.1093/nar/gku1216.

Bernt M., Donath A., Jühling F., Externbrink F., Florentz C., Fritzsch G., Pütz J., Middendorf M., Stadler PF. 2013. MITOS: Improved de novo metazoan mitochondrial genome annotation. *Molecular Phylogenetics and Evolution* 69:313–319. DOI: 10.1016/j.ympev.2012.08.023.

Biomatters 2016. Geneious. Version 9.1.4. [Accessed 2016 Oct 1]. Available from: http://www.geneious.com.

Bolger AM., Lohse M., Usadel B. 2014. Trimmomatic: a flexible trimmer for Illumina sequence data. *Bioinformatics* 30:2114–2120. DOI: 10.1093/bioinformatics/btu170.

Boratyn GM., Camacho C., Cooper PS., Coulouris G., Fong A., Ma N., Madden TL., Matten WT., McGinnis SD., Merezhuk Y., Raytselis Y., Sayers EW., Tao T., Ye J., Zaretskaya I. 2013. BLAST: a more efficient report with usability improvements. *Nucleic Acids Research* 41:W29–W33. DOI: 10.1093/nar/gkt282.

Bushnell B. 2016. BBMap. Version 36.02. [Accessed 2016 Oct 1]. Available from: http://sourceforge.net/projects/bbmap.

Camacho C., Coulouris G., Avagyan V., Ma N., Papadopoulos J., Bealer K., Madden TL. 2009. BLAST+: architecture and applications. *BMC Bioinformatics* 10:421. DOI: 10.1186/1471-2105-10-421.

Capella-Gutiérrez S., Gabaldón T. 2013. trimAl. Version 1.4.rev15. [Accessed 2016 Oct 1]. Available from: https://github.com/scapella/trimal.

Capella-Gutiérrez S., Silla-Martínez JM., Gabaldón T. 2009. trimAl: a tool for automated alignment trimming in large-scale phylogenetic analyses. *Bioinformatics* 25:1972–1973. DOI: 10.1093/bioinformatics/btp348.

Edgar RC. 2004. MUSCLE: multiple sequence alignment with high accuracy and high throughput. *Nucleic Acids Research* 32:1792–1797. DOI: 10.1093/nar/gkh340.

Free Software Foundation 2012. GNU Awk . Version 4.0.1. [Accessed 2016 Oct 1]. Available from: https://www.gnu.org/software/gawk.

Free Software Foundation 2014. GNU Grep. Version 2.16. [Accessed 2016 Oct 1]. Available from: https://www.gnu.org/software/grep.

Haig SM., Mullins TD., Forsman ED., Trail PW., Wennerberg L. 2004. Genetic identification of spotted owls, barred owls, and their hybrids: legal implications of hybrid identity. *Conservation Biology* 18:1347–1357.

Hanna ZR., Henderson JB., Wall JD., Emerling CA., Fuchs J., Runckel C., Mindell DP., Bowie RCK., DeRisi JL., Dumbacher JP. 2017. Northern spotted owl (*Strix occidentalis caurina*) genome: divergence with the barred owl (*Strix varia*) and characterization of light-associated genes. *Genome Biology and Evolution* evx158. DOI: 10.1093/gbe/evx158.

Henderson JB., Hanna ZR. 2016a. BLATq. Version 1.0.2. Zenodo. DOI: 10.5281/zenodo.61136.

Henderson JB., Hanna ZR. 2016b. excerptByIDs. Version 1.0.2. Zenodo. DOI: 10.5281/zenodo.61134.

Johnson M., Zaretskaya I., Raytselis Y., Merezhuk Y., McGinnis S., Madden TL. 2008. NCBI BLAST: a better web interface. *Nucleic Acids Research* 36:W5–W9. DOI: 10.1093/nar/gkn201.

Katoh K. 2016. MAFFT: a multiple sequence alignment program. Version 7.305b. [Accessed 2016 Oct 1]. Available from: http://mafft.cbrc.jp/alignment/software.

Katoh K., Misawa K., Kuma K., Miyata T. 2002. MAFFT: a novel method for rapid multiple sequence alignment based on fast Fourier transform. *Nucleic Acids Research* 30:3059–3066. DOI: 10.1093/nar/gkf436.

Katoh K., Standley DM. 2013. MAFFT Multiple Sequence Alignment Software Version 7: Improvements in Performance and Usability. *Molecular Biology and Evolution* 30:772–780. DOI: 10.1093/molbev/mst010.

Kearse M., Moir R., Wilson A., Stones-Havas S., Cheung M., Sturrock S., Buxton S., Cooper A., Markowitz S., Duran C., Thierer T., Ashton B., Meintjes P., Drummond A. 2012. Geneious Basic: An integrated and extendable desktop software platform for the

organization and analysis of sequence data. *Bioinformatics* 28:1647–1649. DOI: 10.1093/bioinformatics/bts199.

Kumar S., Stecher G., Tamura K. 2016. MEGA7: Molecular Evolutionary Genetics Analysis version 7.0 for bigger datasets. *Molecular Biology and Evolution* 33:1870–1874. DOI: 10.1093/molbev/msw054.

Kumar S., Tamura K., Nei M. 1994. MEGA: Molecular Evolutionary Genetics Analysis software for microcomputers. *Computer applications in the biosciences : CABIOS* 10:189–191. DOI: 10.1093/bioinformatics/10.2.189.

Li H. 2013a. Aligning sequence reads, clone sequences and assembly contigs with BWA-MEM. *arXiv:1303.3997 [q-bio]*.

Li H. 2013b. bioawk. Version 1.0. [Accessed 2016 Oct 1]. Available from: https://github.com/lh3/bioawk.

Li H., Handsaker B., Marshall J., Danecek P. 2015. Samtools. Version 1.3 with HTSlib 1.3.1. [Accessed 2016 Oct 1]. Available from: http://www.htslib.org.

Li H., Handsaker B., Wysoker A., Fennell T., Ruan J., Homer N., Marth G., Abecasis G., Durbin R., Subgroup 1000 Genome Project Data Processing. 2009. The Sequence Alignment/Map format and SAMtools. *Bioinformatics* 25:2078–2079. DOI: 10.1093/bioinformatics/btp352.

Luo R., Liu B., Xie Y., Li Z., Huang W., Yuan J., He G., Chen Y., Pan Q., Liu Y., Tang J., Wu G., Zhang H., Shi Y., Liu Y., Yu C., Wang B., Lu Y., Han C., Cheung DW., Yiu S-M., Peng S., Xiaoqian Z., Liu G., Liao X., Li Y., Yang H., Wang J., Lam T-W., Wang J. 2012. SOAPdenovo2: an empirically improved memory-efficient short-read de novo assembler. *GigaScience* 1:18. DOI: 10.1186/2047-217X-1-18.

Morgulis A., Coulouris G., Raytselis Y., Madden TL., Agarwala R., Schäffer AA. 2008. Database indexing for production MegaBLAST searches. *Bioinformatics* 24:1757–1764. DOI: 10.1093/bioinformatics/btn322.

NCBI Resource Coordinators 2015. Database resources of the National Center for Biotechnology Information. *Nucleic Acids Research* 43:D6–D17. DOI: 10.1093/nar/gku1130.

Omote K., Nishida C., Dick MH., Masuda R. 2013. Limited phylogenetic distribution of a long tandem-repeat cluster in the mitochondrial control region in *Bubo* (Aves, Strigidae) and cluster variation in Blakiston's fish owl (*Bubo blakistoni*). *Molecular Phylogenetics and Evolution* 66:889–897. DOI: 10.1016/j.ympev.2012.11.015.

Pearson WR., Lipman DJ. 1988. Improved tools for biological sequence comparison. *Proceedings of the National Academy of Sciences* 85:2444–2448.

Quinlan AR., Hall IM. 2010. BEDTools: a flexible suite of utilities for comparing genomic features. *Bioinformatics* 26:841–842. DOI: 10.1093/bioinformatics/btq033.

Ruby JG. 2014. PRICE. Version 1.2. [Accessed 2016 Oct 1]. Available from: http://derisilab.ucsf.edu/software/price.

Ruby JG., Bellare P., DeRisi JL. 2013. PRICE: Software for the Targeted Assembly of Components of (Meta) Genomic Sequence Data. *G3: Genes|Genomes|Genetics* 3:865–880. DOI: 10.1534/g3.113.005967.

Tamura K., Kumar S. 2002. Evolutionary Distance Estimation Under Heterogeneous Substitution Pattern Among Lineages. *Molecular Biology and Evolution* 19:1727–1736.

Tamura K., Nei M. 1993. Estimation of the number of nucleotide substitutions in the control region of mitochondrial DNA in humans and chimpanzees. *Molecular Biology and Evolution* 10:512–526.

Zhang Z., Schwartz S., Wagner L., Miller W. 2000. A Greedy Algorithm for Aligning DNA Sequences. *Journal of Computational Biology* 7:203–214. DOI: 10.1089/10665270050081478.
